# Knowledge, Attitude, and Utilization of Traditional Medicine among the Communities of Merawi Town, Northwest Ethiopia: A Cross-Sectional Study

**DOI:** 10.1155/2015/138073

**Published:** 2015-10-05

**Authors:** Samuel Masresha Wassie, Leul Lisanework Aragie, Belaynew Wasie Taye, Laychiluh Bantie Mekonnen

**Affiliations:** ^1^School of Medicine, College of Medicine and Health Sciences, Bahir Dar University, P.O. Box 79, Bahir Dar, Ethiopia; ^2^School of Public Health, College of Medicine and Health Sciences, Bahir Dar University, P.O. Box 79, Bahir Dar, Ethiopia

## Abstract

*Background*. In Ethiopia, up to 80% of the population use traditional medicine for primary health care. Studies on the current knowledge and practices of communities in the era of modern health care expansion are lacking. Therefore, this study is aimed at assessing the knowledge, attitude, and practice of traditional medicine among communities in Merawi town. *Methods*. A descriptive cross-sectional study was carried out among 403 residents of Merawi town. A systematic random sampling was used to select households. Data was collected through house to house interview. *Results*. 392 out of 403 questionnaires were analysed. Among the participants, 220 (56.1%) were female. The mean (±s.d.) age of the participants was 32.5 (±12.4) years. Nearly two-thirds, 241 (61.5%), of study participants have good knowledge about traditional medicines. Three-quarters of participants prefer modern medicine to traditional drugs. 70.9% of participants had the experience of personal use of traditional therapies. *Conclusions*. The population in Merawi has good knowledge with high acceptability and use of traditional medicine. The main reasons for high acceptability and practice were cultural acceptability, lesser cost, and good outcome of traditional medicine.

## 1. Introduction

Traditional medicine (TM) refers to health practice, approach, knowledge, and beliefs incorporating plant, animal, and mineral based medicines, spiritual therapies, manual techniques, and exercises applied singularly or in combination to treat, diagnose, and prevent illness or maintain wellbeing [1].

The widespread use of TM has resulted in traditional health care becoming a lucrative, multinational business. Billions of US dollars are spent annually on traditional medicine in many developed countries. For example, in 2012, 32 billion dollars were spent in the United States of America on dietary supplements, an amount expected to increase to 60 billion dollars in 2021 [2]. The World Health Organization estimates that the global market of traditional medicine is approximately US $83 billion annually [3]. Traditional medicines also contribute to the development of pharmaceutical treatments. As much as one-third to one-half of pharmaceutical drugs was originally derived from plants [4]. Some prominent examples including digitalis, morphine, quinine, and vinca alkaloids were obtained from plant sources [5].

Traditional medicine does more than providing raw materials for pharmaceuticals; holders of traditional knowledge often have valuable knowledge for new drug development. Traditional knowledge can provide valuable guidance in selecting and obtaining plant material of potential therapeutic interest. Bioactive compounds derived from currently used herbal medicines are more likely to have minimal toxicity, and a long history of clinical use suggests that herbal medicine may be clinically effective. Plant-derived compounds used as drugs are generally used in ways that correlate directly with their traditional uses as plant medicines [6].

Countries in Africa, Asia, and Latin America use TM to help meet some of their primary health care needs [7–9]. Despite western medicine becoming more widespread in Ethiopia, Ethiopians tend to rely more on TM. Modern health services remain concentrated in urban areas and have failed to keep pace with the growing population, keeping health care access out of reach for most Ethiopians living in rural Ethiopia. Because TM is culturally entrenched, accessible, and affordable, up to 80% of the Ethiopian population relies on traditional remedies as a primary source of health care [7, 10].

Some studies have also revealed that Ethiopians use TM due to lack of access to modern health care facilities. However, the current Ethiopian health care system is a primary health care focused system which improves access to modern medicine more than ever. But, both rural and urban populations continue to use traditional medicine. The reason behind this was found to be the cultural acceptability of TMPs [11].

Although traditional medicine plays an important role in Ethiopian society, knowledge about the extent and characteristics of traditional healing practices and practitioners is limited and has frequently been ignored in the national health system. This is also true in Merawi where such study on traditional medical practice (TMP) was not conducted in the past [7, 8, 12]. Based on the above insight the study focused mainly on identifying the knowledge, perceptions, and practice of TM through cross-sectional study in Merawi town, northwest Ethiopia.

By studying the knowledge, attitude, and practices of traditional medicine in Merawi town, our study will provide important data for the town administration and Amhara Regional Health Bureau to take appropriate controlling measures regarding the quality and safety of the practices. The study will also provide the baseline data for researchers for further investigations to determine the determining factors of TM uses.

## 2. Method

### 2.1. Study Design

A community based cross-sectional study design was used to assess knowledge, attitude, and utilization of the community towards traditional medicine, in Merawi town, 2015.

### 2.2. Study Setting

Study was conducted in Merawi town. Merawi is the capital of Mecha District found in northwest Ethiopia. It is found at a distance of 36 km from Bahir Dar. Its climatic condition is “Woina Dega.” Currently, it has a total population of 40,635 people of which 18,597 (45.77%) are males and 22,038 (54.23%) females. There are about four private clinics, one public health center, and a newly constructed standardized hospital which will be officially opened soon for public service.

### 2.3. Population

All the households in Merawi town were the source population of the study. The study population included individuals aged greater than 18 years and living for at least six months in the town. The sampling units were households, while the study units were adult individuals available in the household during the interview, preferably the woman, where more than one adult were found.

### 2.4. Sample Size Calculation

Sample size was calculated based on the prevalence of knowledge, attitude, and practice based on the following assumptions: *P* = 80% prevalence of TM users in Ethiopia [7], *Z*  (1.96) is the value under standard normal table for confidence level of 95%, margin of error (*d*) = 4%, and using the formula for estimation of single population proportions *n* = *Z* ^2^ *P*(1 − *P*)/*d* ^2^. And adding a nonresponse rate of 5%, the final sample size became 403 adults. *n* is the required sample of the study.

### 2.5. Sampling Procedure

A systematic random sampling technique was used to select households. The first household was selected from the list of initial 6 households by lottery method. Then every 6th household was selected and adults in the household were interviewed. In the presence of more than one adult the woman was interviewed as women took the highest responsibility in the care of family members. In the absence of woman, the husband or other adults were interviewed.

### 2.6. Data Collection Procedure

Data were collected using structured interviewer administered questionnaire adapted from standardized questionnaires used by international organizations, national studies such as Demographic and Health Survey, and published articles in peer-reviewed journals. Data were collected by trained data collectors using face-to-face interview.

#### 2.6.1. Data Quality Control

Intensive training was provided to data collectors about data collection techniques. Detail orientation was given to the data collectors about the study before data collection procedure starts. A translation of data collection instruments into local language was done. A pilot test was done on 40 (10% of the sample population) households to validate consistency of the questions and data collection tool.

### 2.7. Study Variables

The outcome variables of the study were knowledge, attitude, and utilization of the community on TMs. The explanatory variables were age of interviewee, monthly family income, educational status, distance from nearby health facility, and membership of community health insurance.

### 2.8. Data Management and Analysis

Data were checked for completeness and consistency and entered into SPSS version 20 by principal investigators, cleaned, and analyzed. The results were presented using simple frequencies with percentages in appropriate tables to display the descriptive part of the result.

Five yes or no questions were asked for each respondent regarding harmful TMs, side effects of TMs, and importance of training about TMs. The number of questions for which the respondent gave correct responses was counted and scored. This score was then pooled together and the mean score was computed to determine the overall knowledge of respondents; respondents who score greater than or equal to the mean value were grouped to have good knowledge and and those who score less than the mean value poor knowledge level. The attitude of the respondents was assessed using eight yes or no questions focusing on the history of training about TM, recommending these methods to the others, effectiveness of methods for applied cases, interest to learn TCM, and choice of training methods.

### 2.9. Ethical Issues

Formal letter of approval was obtained from the Ethical Review Board of College of Medicine and Health Science to Merawi town administration. Each participant of the study was informed about confidentiality. Each participant of the study agreed to participate voluntarily. Participants were allowed to discontinue the interview when they needed. All participants of the study declared their willingness to participate and approved by their verbal consents.

## 3. Results

### 3.1. Sociodemographic Characteristics

A total of 392 respondents, with a response rate of 97.3%, were studied. Among the participants, 220 were females (56.1%) and the rest were males. Ages of participants ranged from 18 to 85 (mean age of the participants was 32.54 ± 12.44 years). Regarding the religion, 286 (73%) of the study participants were follower of Orthodox Christianity followed by Muslims 85 (23.7). From the total respondents about 75 (19.1%) were government employees. 198 (50.54%) participants have family size of 3 to 5 family members. With respect to income 83 (21.8%) of the respondents reported that their annual incomes were above the mean annual income (33318.49 birr). Large number of respondents, 115 (29.3%), cannot write and read while 113 (28.8%) respondents attended secondary school. Two 208 (53.1%) of the respondents were married (Table 1).

### 3.2. Knowledge about Traditional Medicine

The mean (±) value knowledge score was 4.4 (±0.97). The data in Table 2 shows that 241 (61.5%) of the study participants were found to have good knowledge about TMs and 151 (39.5%) had poor knowledge level. Most of the respondents 352 (89.8%) responded that there is no harmful traditional practice. However, 379 (96.7%) respondents knew that exposure to nonsterile material could result in HIV infection to the users (Table 2).

### 3.3. Attitude towards Traditional Medicine

**Figure 1:**
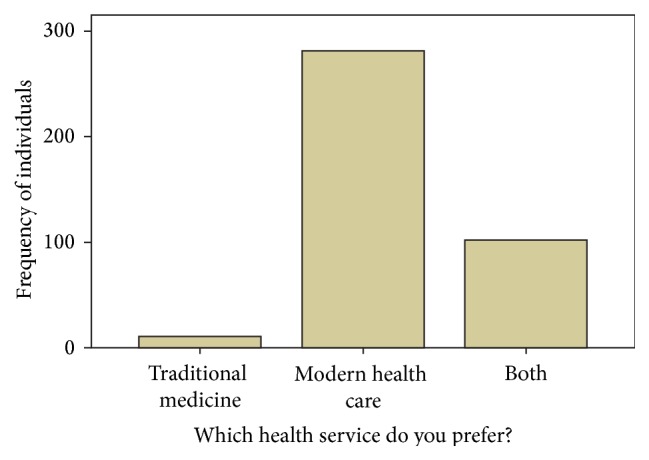
Preference of health care system and attitude of traditional medicine among Merawi residents, April 2015, utilization of traditional medicines.

Two hundred eighty-one (71.7%) of the study participants prefer to use modern health service rather than traditional medicines (Figure 1). Regarding the effectiveness of the TMs, 275 (70.2%) participants do not agree about effectiveness of TMs compared to modern health care service after use. Only 75 (19.1%) participants recommend using TM therapy for others. Among the participants, 232 (59.2%) believe TMs are still accepted in the community and 115 (49.5%) respondents agree that the reason is cultural acceptability and 42.2% account the good outcome of TM after they use them.

Sixty nine (17.6%) participants have previously attended education or training about the benefits and adverse effects of traditional medicine. Majority, 354 (90.3%), of the respondents strongly felt that they want training about these issues (Table 2) and 210 (53.6) of them prefer the public awareness creation about TMs.

About 278 (70.9%) participants had used different types of herbal medicines either by themselves or visited traditional healer at least once in their lifetime for treatment. Of those respondents, 171 (64%) had used only herbal medication of different type as mode of treatment for various illnesses (Table 4). Furthermore, out of herbal medication users, 98.8% has used herbal medication for 6 months. Only 22 (5.6%) respondents had history of concurrent use of modern medicines along with TMs. Aspirin, Paracetamol, Amoxicillin, and antacids are the commonest drugs used with TMs. Eighty-nine (22.7%) respondents experienced adverse effects in their family members due to traditional medicine therapy. The reported adverse effects include bleeding, abortion, visual loss, tetanus, jaundice, fistula, gastritis, psychosis, exacerbation of illness, paralysis, and even death (Table 3).

### 3.4. Factors Associated with Traditional Medicine Use

Association between independent variables and KAP scores on TMs was calculated using Pearson's Chi square. There was significant association between KAP scores and age (*P* = 0.05), specially with age group between 18 and 28 and 29 and 38 (*P* = 0.02 and 0.004, resp.). Educational status was also significantly associated with KAP scores (*P* = 0.00). Moreover, occupation (*P* = 0.00) and effectiveness of TMs (0.002) were found to be associated with KAP score on TMs (Table 4).

## 4. Discussion

The prevalence of TM use in our study is 70.9%. Our finding is somewhat lower when compared to another study conducted in Ethiopia which states that the prevalence of traditional medicine use is 80% [13]. This is probably due to the sample size difference where the later takes large sample size. 70.9% prevalence is still high and this can be ascribed to the fact that majority of people in our study (59.2%) believe that traditional medicine is still acceptable in the communities of Merawi town. The wider acceptability of traditional medicine in the town is because of cultural acceptability, easy accessibility, and affordability of traditional medicine compared to modern medicines and facilities [10, 14].

Among the practiced traditional medicine the commonest are herbal medication 64%, uvulectomy 20.1% [15], and spiritual healing (7.6%). 22.7% of the population experienced adverse effects after the use of traditional medicine and the commonest are bleeding, fistula, psychosis, and exacerbation of illness. This is different from population based study conducted in northern Ethiopia during 2005; the prevalence rates of uvulectomy and milk teeth extraction were 89% and 58%, respectively [7]. This shows that as the level education of mother increased the use of harmful traditional medicine practice decreased, in addition to the fact that TMs vary together with cultural diversity of the country.

Our study revealed that 61.5% of the population has good knowledge about traditional medicines which is different from the study conducted in Lagos Nigeria in which 44.7% of the population had good knowledge [16]. The discrepancy in the results between the two studies might be associated with mainly the time gap in which the studies are conducted which changes the awareness of the population about traditional medicine and coverage of modern health care. The socioeconomic differences between the two towns (Lagos is the capital city of Nigeria, while Merawi is a Woreda town) might also contribute to the observed differences.

Our study also indicated that more than two-thirds (82.4%) of the participants had no previous formal education on the benefits and adverse effects of traditional medicine, but majority (90.3%) of participants showed interest to acquire education in this regard. Moreover, 53.6% of the participants best prefer the public health education as a means of awareness creation. These findings show that most of the participants in the study had positive attitude toward traditional medicine.

The participants have reported that they use many plant products for different disorders. The people prepare the plants in different dosage forms (liquid, solid, and gaseous forms) and administer them by mixing with water, tea, egg, and honey or without any mixing. Different studies also reported similar practices [17, 18]. The plant preparations are mainly used once daily for few days (ranges from 1 day to 6 months). Most commonly used routes of administration are oral, topical, and inhalational routes of administration. Previous studies reported that oral, dermal, and nasal routes are the three most commonly used routes of administration which is in line with our study [18–20].

Many diseases such as headache, cough, peptic ulcer disease, asthma, cold, skin disease, hypertension, and others are reported to be treated with the different plant preparations in our study. Leaves, stems, and seeds were mainly used for treatment. The plants were obtained from home garden, market, or traditional medicine practitioner. Other studies done in different parts of Ethiopia have reported that leaves of the plants are the most commonly used parts of the plants for treatment of similar disorders/diseases [21–23].

In our study, we found that the use of traditional medicine was significantly associated with the age of the population; particularly age groups of 18–28 and 29–38 were highly associated with the level of traditional medicine use with *P* value < 0.02 and 0.004, respectively. Educational status and occupation of the participants were found to be highly associated with the use of traditional medicine (*P* value = 0). Religion, family size, annual income, and marital status were found to have no association with use of traditional medicine.

## 5. Conclusions

The population has good knowledge of the TM. The acceptability and prevalence of traditional medicine in the town are high and this is related to the cultural acceptability, easy accessibility, and affordability of TM. Lack of access to modern health service is also another factor which contributed to the high prevalence of TM use by the respondents. Herbal medication use is the most common type of TM practiced in the population. Herbal medications were used by the community to treat headache, cough, peptic ulcer disease, asthma, cold, skin disease, hypertension, and other related diseases. The herbal products which are mainly leaves, stems, and roots were obtained mainly from home garden. Bleeding, fistula, and psychosis are the major adverse effects of herbal medications reported by the population. Most of the population show interest to have education regarding the benefit and adverse effects of TM. Age group, economic status, and educational status were found to be highly associated with high prevalence of TM use, whereas family size, annual income, and religion were found to have no association with TM use.

## Figures and Tables

**Table 1:** Sociodemographic characteristics of respondents, Merawi town, April 2015.

Characteristic	Number of persons	Percent
Sex		
Male	172	43.9
Female	220	56.1
Age of respondent (years)		
18–28	187	47.7
29–38	95	24.3
39–48	66	16.8
>49	44	12.2
Occupation of respondent		
Farmer	12	3.0
Merchant	90	23
Government employee	77	19.61
Student	58	14.8
Self-employed	23	5.8
Unemployed	28	7.1
Housewife	74	18.9
Others	30	7.7
Annual income		
2,400–50,000	199	50.9
50001–100,000	34	8.8
>100,000	5	1.3
Marital status of respondent		
Married	208	53.2
Never married	147	37.6
Divorced	10	2.6
Widowed	26	6.6
Education of respondent		
Illiterate	115	29.3
Primary school	89	22.7
Secondary school	113	28.8
Higher education	14	3.6

**Table 2:** Knowledge of Merawi residents of traditional medicines, April 2015.

Characteristics	Frequency	Percentage
There is no harmful traditional medicine		
True	352	89.8
False	40	10.2
Traditional medicines have no adverse effect		
True	350	89.3
False	42	10.7
Health education about risks and benefits of traditional medicines is important		
True	354	90.3
False	38	9.7
Exposure to nonsterile material will expose to HIV		
True	379	96.7
False	13	3.3
Traditional medicines are more effective and safer than modern health services?		
True	102	26
False	290	84

**Table 3:** Medicinal plant used for the treatment of human diseases: local name, scientific name, disease treated, part(s) used, dosage form, method of preparation, administration route, duration of use, and source of plant.

Local name	Scientific name	Disease treated	Part of the plant	Dosage form used	Rout of administration	Method of preparation	Duration of treatment	Frequency	Source of the plant
Damakese	*Ocimum lamiifolium *Hochst.	Unexplained headache and fever	Leaf	Fluid	Mouth	Tea	1 day	Once daily	Home garden

Tenadam	*Ruta chalepensis *L.	Unexplained headache and fever	Leaf	Fluid	Mouth	Coffee/tea	1 day	Once daily	Home Garden, market
		Cold	Leaf	Fluid	Mouth	Tea/coffee	3 days	Twice daily	Home garden

Tikur azmud	*Nigella sativa*	Peptic ulcer disease	Seed	Fluid	Mouth	Water	5 days	Once daily	Market
		Cold	Seed	Fluid	Mouth	Tea	3 days	Twice daily	Market

Haregresa	*Zehneria scabra*	Cough	Leaf	Smoke	Nose	Boiling with water	5 days	Twice daily	Traditional healers

Tunjit	*Otostegia fruticosa*	Cold	Leaf	Smoke	Nose	Burning with fire	3 days	Once daily	Market

Kundo berbere	*Piper nigrum*	Tonsil	Seed	Flour	Mouth	Tea	3 days	Once daily	Market

Gesho	*Rhamnus prinoides *L.	Tonsil	Leaf	Powder	Mouth	Tea	1 day	Twice daily	Traditional healers

Zinjibl	*Zingiber officinale *Rosc.	Abdominal pain	Seed	Powder	Mouth	Water	1 day	Once daily	Market
		Cold	Seed	Fluid	Mouth	Tea	4 days	Thrice daily	Market
		Fever	Stem	Powder	Mouth	Tea	1 day	Once daily	Market

Girawa	*Vernonia amygdalina *Del.	Unexplained headache and fever	Leaf	Fluid	Mouth	Coffee	3 days	Twice daily	Home garden

Key shinkurt	*Allium cepa *L.	Asthma	Root	Fluid	Mouth	Water	5 days	Once daily	Market

Tekeze	*Andrachne aspera*	Snake bite	Stem	Fluid	Mouth	Water	10 days	Twice daily	Traditional healers

Bisana	*Croton macrostachyus *	Skin damage	Leaf	Fluid	Topical	Egg	14 days	Once daily	Traditional healers
		Tinea versicolor	Leaf	Exudates	Topical	Alone	3 days	Once daily	Traditional healers

Nech bahirzaf	*Eucalyptus globulus*	Fever	Leaf	Smoke	Nose	Boiling in water	2 days	Once daily	Traditional healers

Zigba	*Podocarpus gracilis*	Acute hepatitis	Leaf	Fluid	Mouth	Water	60 days	Once daily	Traditional healers

Abish	*Trigonella foenum-graceum*	Peptic ulcer disease	Seed	Fluid	Mouth	Water	5 days	Once daily	Market

Tosegn	*Thymus schimperi*	Hypertension	Leaf	Fluid	Mouth	Tea	6 months	Once daily	Market/home garden

**Table 4:** Relationships between KAP scores about TMs and some key independent variables among study respondents of Merawi town, April 2014.

Variable	*χ* ^2^	*P* value
Sex	2.47	0.118
Age	33.05	0.05
Annual income of the family	4.25	0.834
Religion	2.01	0.919
Occupation	51.05	0.00
Educational status	37.38	0.00
Family size	30.98	0.056
Effectiveness	16.49	0.002
Marital status	5.84	0.212
